# Internalizing and externalizing problems, depression, and self-esteem in non-detained male juvenile offenders

**DOI:** 10.1186/1753-2000-7-7

**Published:** 2013-02-27

**Authors:** Daniela Imbach, Marcel Aebi, Christa Winkler Metzke, Cornelia Bessler, Hans Christoph Steinhausen

**Affiliations:** 1 Forensic Child and Adolescent Psychiatry, Department of Child and Adolescent Psychiatry, University of Zurich, Zurich, Switzerland; 2 Clinical Psychology and Epidemiology, University of Basel, Basel, Switzerland; 3 Research Unit of Child and Adolescent Psychiatry, Psychiatric Hospital, Aalborg University Hospital, Aalborg, Denmark

**Keywords:** Juvenile offenders, Internalizing and externalizing problems, Depression, Self-esteem

## Abstract

**Background:**

High rates of mental disorders have been found in detained juvenile offenders, whereas the role of psychopathology in non-detained offenders is less clear. Therefore, the present study compared psychopathology in male non-detained delinquent juveniles and two matched samples from the community and an adolescent psychiatric clinic.

**Methods:**

125 male adolescents aged 11 to 19 years (m = 16.2 years, SD = 1.5 years) from an outpatient adolescent forensic clinic were compared to a community sample from the Zurich Adolescent Psychology and Psychopathology Study (ZAPPS) and a referred sample from a psychiatric clinic matched for age and nationality. All subjects responded to questionnaires measuring internalizing and externalizing problems, depressive symptoms and self-esteem.

**Results:**

The sample of non-detained juvenile offenders showed similar rates of self-reported internalizing and externalizing problems when compared to the community sample, whereas the clinic sample displayed an increased rate of various disturbances. Similar results were found also for self-esteem. In agreement with these findings, non-detained juvenile offenders less frequently had a psychiatric diagnosis after full clinical assessment when compared to the clinical sample. However, a diagnosis of conduct disorders and a lower IQ range was found more frequently in non-detained juvenile offenders. Offenders with serious delinquent acts and involving weapons showed higher depression scores than the rest of the offenders.

**Conclusion:**

In non-detained assessment situations before court examination, juvenile offenders present rather normal behaviour. Their lack of awareness of potential behavioural problems should be considered during assessment and treatment of this group of offenders.

## Introduction

There is clear evidence that incarcerated juveniles show high rates of psychiatric disorders and comorbidity [1-6]. In particular, high rates of conduct disorders, ADHD, anxiety, depression, substance abuse and PTSD were found. Even after excluding common conduct disorders, delinquent adolescents still show high rates of other mental disorders. Compared to community and clinical samples, they display higher rates of internalizing and externalizing disorders [2,7,8]. However, according to Vermeiren [5], findings in incarcerated juveniles cannot be transferred to non-detained offenders. Currently, only a few studies have evaluated the frequencies of mental disorders in general in this specific group of non-detained juveniles [2,5,9]. An increased rate of conduct disorders, substance abuse and depressive symptoms were found in two of these studies [10,11].

Among various mental disorders, depression and affective disorders play a particular role in juvenile offenders. Ritakallio et al. [9] found a robust association between self-reported delinquency and depression. The strongest associations with depression were identified for frequent vandalism and violent behaviour. Depression increased with the frequency of delinquent behaviour in boys as well as in girls. Mood disorders were frequently seen both in incarcerated juvenile delinquents and in juveniles with mixed detention situations with prevalence rates varying between 10 to 78% [5,12]. Furthermore, high scores of adjustment disorders with depressed mood were also observed in outpatient samples [10] and high rates of depressive symptoms based on the Beck’s Depression Inventory [13] were reported [14,15]. In addition, Howard [16] found that juveniles with more serious offenses had higher levels of depression in self-reports. However, this relation was not replicated in the study by Alessi, McManus, Grapentine & Brickman [17].

Among various personality features, self-esteem plays a major role due to its close association with various kinds of psychopathologies and delinquencies. There are contradictory findings on the association of self-esteem and delinquency with some studies finding no relation between these two variables [8,18,19], and others reporting a negative association between self-esteem and delinquency [20-23]. It has also been postulated that low self-esteem fosters delinquency and delinquency vice-versa may enhance self-esteem [24]. Most of these studies used self-reports to identify delinquency. Furthermore, a grandiose sense of self-worth has been postulated as a feature of psychopathy in juvenile offenders and is reflected in the Hare Psychopathy Checklist: Youth Version [25].

In addition to focusing on psychopathology and personality features in both incarcerated and non-detained juvenile delinquents there is also a need to more clearly specify the type of offenders to whom the observed abnormal features most strongly apply. This more recent approach has made a distinction between sexual and non-sexual offenders. In their recent review, Van Wijk, Vermeiren, Loeber, ‘t Hart-Kerkhoffs, Doreleijers & Bullens [26] suggested that sexual offenders are more likely to display internalizing problems than non-sexual offenders and that findings regarding externalizing problems are less consistent in this group.

The first aim of the present study was the comparison of internalizing and externalizing problems, depressive symptoms and self-esteem among male non-detained juvenile offenders, a male community group, and a clinical group of male adolescents attending a mental health service. More specifically, we assumed that based on self-reports the delinquent sample would show (a) higher levels of internalizing and externalizing problems and depressive symptoms and a lower self-esteem than the community sample and (b) similar scores in these measures when compared to the clinical sample. The second aim of the study was to compare the frequencies of psychiatric diagnoses after full clinical assessment. It was expected that the frequencies of diagnoses would be similar in the forensic and the clinical sample. Finally, the third aim of the present study was to analyze the association of specific offense characteristics such as sexual offenses vs. non-sexual offenses as well as the severity of crimes on internalizing and externalizing problems, depressive symptoms and self-esteem.

## Methods

### Subjects

The present study is based on three male samples, namely a forensic sample with 125 delinquent juveniles and two matched samples from a mental health service (clinical sample) and a community survey matched for age and nationality. In each sample, there were N = 12 participants who were aged 11–14 years, N = 83 aged 15–17 years and N = 30 aged 18–19 years. In each sample, the age was 16.2 (SD = 1.5) years and N = 87 participants were of Swiss origin whereas N = 38 were migrants living in Switzerland.

Originally, the forensic sample included all offenders who had been assessed on judicial order at the Forensic Child and Adolescent Outpatient Division, Department of Child and Adolescent Psychiatry, University of Zurich, Switzerland during the years from 2005 to 2008. Of the initial 199 adolescents, 43 were excluded due to missing values (>20%) in one or more questionnaire (see below). An additional 31 individuals were excluded because of either female gender or incarceration. Thus, the final sample included 125 young male delinquents. A total of 67 (53.6%) adolescents in the forensic sample were repeated offenders at the time of the assessment. In terms of the type of delinquency, 43 (34.4%) had been involved in violent offenses, 50 (40%) in property crimes, 54 (43.2%) in one or more current sex offenses and 41 (32.8%) in another type of delinquency. There were 67 (62.6%) multiple offenders.

The community sample was based on the original sample of the Zurich Epidemiological Study of Child and Adolescent Psychopathology (ZESCAP) in 1994 with 1964 pupils aged 6 to 17 years, living in the canton of Zurich, Switzerland [27]. From this cohort, a subsample of 1110 adolescents was repeatedly assessed in 1997, 2001, and 2005 within the Zurich Adolescent Psychology and Psychopathology Study (ZAPPS) [27,28]. For the comparison with the forensic sample, a matched sample of 125 male individuals from the 1997 wave of assessments was randomly drawn.

The clinic sample consisted of patients attending the Child and Adolescent Psychiatric Service (CAPS) of the Canton of Zurich, Switzerland, during the years 1994 to 2010. Participants included both outpatients and inpatients. From this cohort of 2428 patients, another matched sample of 125 individuals was randomly drawn.

The assessment of the forensic sample took place under the order of the referring legal prosecutors and included informed consent from the adolescents. The community sample was collected in the nineties of the last century prior to the existence of an ethical committee in the region of the study. The patient sample came from a large child and adolescent service where parents and adolescents provided informed consent for assessment and treatment.

The ZAPPS community study was approved by the local school authorities of the government of the Canton Zurich, Switzerland, at a time when there was not yet existing an official ethical commitee for scientific studies. In addition, the study was based on informed consent of all participating adolescents and their parents. In agreement with the guidelines of the local ethical committee of the Canton Zurich, Switzerland, data from the clinical and forensic samples was collected as a part of the clinical assessment and did not need a special approval by the local ethical committee. When attending the local services, all juveniles and their parents of the clinical and forensic samples provided written consent that their data can be used in an anonymised form for research purposes.

### Measures

#### Youth self-report (YSR)

The study used the Swiss version [29] of the Youth Self-Report (YSR) [30] which consists of 113 items leading to the following primary subscales: social withdrawal, somatic complaints, anxiety/depression, social problems, thought problems, attention problems, delinquent behaviour, and aggressive behaviour. Two second-order scales reflecting internalizing and externalizing and a total problem score can be calculated. The time frame of symptoms includes the past 6 months. Reliability and validity have been shown to be good both for the original version [30] and the Swiss version [29] of the YSR.

#### Center for epidemiological studies depression scale (CES-D)

The Swiss adaptation [31] of the German version [32] of the CES-D [33] was used for the measurement of adolescent depression. The time frame for reporting symptoms according to the instructions of the questionnaire was the week prior to filling out the questionnaire. A total score of the 20 items was calculated. Adequate reliability of the Swiss version has been documented [31].

#### Self-esteem

The translated version of the 10-item scale for the measurement of self-esteem by Rosenberg [24] was used in the present study. This scale has also been shown to have adequate reliability [28].

#### Clinical diagnosis

Consensus diagnoses were obtained for each participant of both the forensic sample and the clinic sample by standardized quality procedures involving the approval of junior clinicians’ diagnoses by senior expert clinicians. Diagnoses were based on the International Classification of Diseases [34] criteria and the entire clinical information available including history, behavioural observation, clinical questionnaires, parent, school and other reports, and psychological testing.

#### Forensic psychiatric documentation system

Each client attending our unit receives a standardized documentation of data including socio-demographic data, developmental and crime history, psychiatric diagnoses, and offense characteristics. To study the impact of the various offenses, the latter were defined by the following variables: sexual vs. non-sexual offending, history of other previous crimes, reoffending with the same type of delinquency, group delinquency vs. individual delinquency, use of weapon, severity of delinquent act (defined by threat of punishment according to the Swiss Penal Code with < = 5 years imprisonment for less severe offenses and >5 years for major delinquency), and use of drugs or alcohol at the time of the offense.

### Statistical analyses

In a first step, multivariate General Linear Models (GLM; SPSS 14.0) were used to compare means of self-reported internalizing and externalizing problems, depression, and self-esteem in the forensic, the community, and the clinical sample. The multivariate approach was used to avoid type 1 error. The models were followed by post hoc comparisons of the means by Scheffè tests. Furthermore, Chi^2^ statistics were used to compare the frequencies of diagnoses in the clinic and the forensic sample. Analyses of offense characteristics within the forensic sample were based on independent t-tests comparing the means of various subgroups.

## Results

As can be seen in Table 1, there were no significant differences in the scores of the various YSR problem subscales between the forensic and the community sample. In particular, there was no significant difference between the forensic and the clinic sample regarding delinquent behaviours. In contrast, the clinic sample differed significantly from the other samples on all subscales except for ‘aggressive behaviours’. The scores of the second-order scale ‘externalizing problems’ were not significantly different among the three samples. However, both the forensic sample and the clinic sample reported significantly higher depression scores on the CES-D scale than the community sample. Regarding self-reported self-esteem, the forensic sample did not differ from the community sample. Both samples showed a significantly higher self-esteem than the clinic sample.

**Table 1:** Comparison of the YSR scores, the CES-D depression score, and the self-esteem score in three samples

	**Forensic sample (N = 125)**		**Community sample (N = 125)**		**Clinic sample (N = 125)**		**df**	**F**	**Post hoc comparisons (Scheffè’test)**
	**A**		**B**		**C**				
	**Mean**	**SD**	**Mean**	**SD**	**Mean**	**SD**			
YSR social withdrawal	2.19	2.23	2.27	1.92	4.20	3.01	2	27.33***	A,B < C
YSR somatic complaints	2.26	2.41	2.15	1.79	3.27	2.62	2	9.07***	A,B < C
YSR anxious/depressed	4.78	4.47	4.44	3.38	7.12	5.14	2	13.81***	A,B < C
YSR social problems	1.58	1.72	1.91	1.96	2.87	2.50	2	12.81***	A,B < C
YSR thought problems	1.63	1.67	2.07	1.89	3.16	2.72	2	16.92***	A,B < C
YSR attention problems	3.94	2.76	3.67	2.45	5.34	2.71	2	14.30***	A,B < C
YSR delinquent behaviours	4.13	2.80	3.76	2.38	5.01	2.91	2	6.99***	B < C
YSR aggressive behaviours	7.94	5.37	7.99	4.62	8.41	5.78	2	.30	
YSR internalizing problems	8.98	7.25	8.59	5.63	13.87	8.21	2	21.35***	A,B < C
YSR externalizing problems	12.07	7.24	11.76	6.28	13.42	7.77	2	1.92	
YSR total score	34.10	18.74	33.42	15.07	45.04	20.55	2	15.94***	A,B < C
CES-D depression	15.98	11.77	9.77	6.45	18.56	12.06	2	23.54***	A,C > B
Self-esteem	26.46	6.26	27.90	5.35	23.95	7.36	2	12.34***	A,B < C

*Note: *Primary YSR subscales: Pillai’s Trace; F = 5.239; df =732/16; p < .001, Second order YSR-scales : Pillai’s Trace; F =10.164; df =744/4; p < .001, YSR-total score, CES-D, and Self esteem: Pillai’s Trace; F = 10.968; df =742/6; p < .001, * = p < .05, ** = p < .01, *** = p < .001.0.

The distribution of psychiatric diagnoses in two samples is shown in Table 2. In the clinic sample a significantly increased presence of at least one psychiatric diagnosis was observed. However, the rates of comorbid diagnoses did not differ significantly between the two groups. When compared to the forensic sample the clinic sample, displayed significantly more schizophrenia spectrum disorders (F20), more mood disorders (F30), more neurotic disorders (F40), and more disorders of psychological development (F80). On the other hand, there were significantly more conduct disorders in the forensic sample but no significant differences between the two samples in the main category of behavioural and emotional disorders (F90). Furthermore, the proportion of adolescents with low intelligence was significantly higher in the forensic sample.

**Table 2:** Distribution of psychiatric diagnoses in the forensic and the clinic sample

	**Forensic sample (N = 125)**	**Clinic sample (N = 125)**	**Chi**^**2**^**-Test**
**Number of diagnoses**	**N (%)**	**N (%)**	
No diagnosis	58 (46.4%)	11 (9.1%)	42.41***
1 diagnosis	45 (36.0%)	84 (69.4%)	27.54***
>1 diagnoses	22 (17.6%)	26 (21.5%)	.59
Psychiatric categories			
Organic disorders (F00-F09)	2 (1.6%)	1 (0.8%)	.34
Substance abuse (F10-F19)	11 (8.8%)	8 (6.4%)	.51
Schizophrenia (F20-F29)	0 (0%)	9 (7.2%)	9.34**
Mood disorders(F30-F39)	8 (6.4%)	22 (17.6%)	7.42**
Neurotic disorders (F40-F48)	7 (5.6%)	28 (22.4%)	14.65***
Physiological disturbances (F50-F59)	0 (0%)	1 (0.8%)	1.00
Personality disorders (F60-F69)	8 (6.4%)	5 (4%)	.73
Development disorders (F80-F89)	0 (0%)	4 (3.2%)	4.07*
Behavioural and emotional disorders	52 (41.6%)	54 (43.2%)	.07
Conduct disorders (F90.1, F91, F92)	46 (36.8%)	30 (24%)	4.49*
Intelligence			
Average (IQ 85–115)	85 (68%)	86 (68.8%)	.02
Above (IQ > 115)	15 (1.2%)	26 (20.8%)	3.60
Below (IQ < 85)	14 (11.2%)	2 (1.6%)	9.68**

Note: * = p < .05, ** = p < .01, *** = p < .001.

Findings based on various subgroups of specific offense characteristics within the forensic sample are presented in Tables 3 and 4. To avoid chance findings, only total scores of the various behaviour variables were included in the analyses. When comparing sexual and non-sexual offenders, the data of eight offenders had to be excluded from the analyses because they had committed sexual as well as non-sexual crimes. Non-sexual offenders showed more internalizing problems than sexual offenders. The number of previous delinquent acts was not significantly related to any behavioural characteristic and there was also no significant association between repeated delinquent activity in the same offending category and any of the dependent variables. The depression score was significantly higher in group delinquents compared to individual delinquents. Delinquents who used weapons were significantly more depressed than those not using weapons. Adolescents with more severe delinquent acts showed higher depression scores and lower self-esteem than those with minor delinquent acts. Finally, the use of drugs at the time of the offense did not have a significant effect on any variable.

**Table 3:** The association of sexual offending, history of other previous crimes, and reoffending with behavioural variables

**Criminal characteristics**	**Sexual offending**			**History of other previous crimes**			**Reoffending**		
	**Mean (SD) (df = 115)**		***t*-Test**	**Mean (SD) (df = 107)**		***t*-Test**	**Mean (SD) (df = 109)**		***t*-Test**
	**Only sex (N = 46)**	**Non-sex (N = 71)**		**No (N = 52)**	**Yes (N = 57)**		**No (N = 77)**	**Yes (N = 34)**	
Internalizing problems YSR	7.3 (5.9)	10.1 (7.8)	2.2*	9.3 (7.2)	10.0 (7.7)	.5	8.9 (7.0)	10.8 (8.4)	1.2
Externalizing problems YSR	10.8 (6.6)	13.2 (7.7)	1.8	11.4 (7.2)	12.8 (7.2)	1.0	11.2 (6.7)	13.7 (7.7)	1.7
CES-D	14.0 (10.9)	17.2 (12.3)	1.5	17.4 (11.7)	16.5 (12.4)	.4	16.0 (12.1)	19.3 (11.4)	1.4
Self-esteem	27.0 (6.2)	26.1 (6.0)	.8	25.8 (7.0)	26.1 (5.6)	.3	25.9 (6.5)	25.9 (6.0)	.0

Note: *p < .05, **p < .01.

**Table 4:** The association of individual vs. group delinquency, use of weapons, severity of delinquent acts, and offense under the influence of drugs with behavioural variables

**Offense characteristics**	**Individual vs. group delinquency**			**Use of weapons**			**Severity of delinquent acts**			**Offense under the influence of drugs**		
	**Mean (SD) (df = 115)**		***t*-Test**	**Mean (SD) (df = 115)**		***t*-Test**	**Mean (SD) (df = 115)**		***t*-Test**	**Mean (SD) (df = 115)**		***t*-Test**
	**Individual (N = 55)**	**Group (N = 62)**		**No (N = 87)**	**Yes (N = 29)**		**Less severe (N = 78)**	**More severe (N = 47)**		**No (N = 86)**	**Yes (N = 22)**	
Internalizing problems YSR	7.8 (6.4)	10.0 (7.9)	1.7	8.5 (6.6)	11.3 (9.3)	1.5	8.4 (6.7)	9.9 (8.1)	1.1	8.8 (6.6)	10.1 (9.2)	.8
Externalizing problems YSR	11.9 (7.4)	12.1 (7.0)	.2	11.4 (7.0)	12.3 (6.7)	.6	12.4 (7.0)	11.5 (7.6)	.6	11.5 (6.9)	13.3 (8.6)	1.0
CES-D	13.6 (10.2)	18.7 (13.0)	2.3*	14.9(10.7)	21.2(14.3)	2.2*	12.9 (9.6)	21.0 (13.4)	3.6**	16.2 (11.8)	13.1 (12.8)	.2
Self-esteem	27.0 (6.1)	25.6 (6.5)	1.2	26.5(6.0)	25.3 (7.3)	.9	27.4 (6.0)	25.0 (6.4)	2.1*	25.9(6.6)	26.8 (6.2)	.6

## Discussion

In the present study, the comparison of self-reports showed no differences in internalizing and externalizing problems in non-detained juvenile offenders and the community sample. However, the forensic sample had significantly more depression symptoms and the clinic sample was significantly more disturbed than the other two samples including lower scores in self-reported self-esteem.

The analysis of clinical diagnoses revealed that half of the forensic sample had no diagnosis, whereas the clinical patients significantly more often had at least one diagnosis. However, co-morbidity rates were not significantly different in the two samples. When comparing clinical diagnoses in the clinic and the forensic sample, the clinic sample displayed more psychopathology in various domains, whereas the forensic sample showed more conduct disorder diagnoses and borderline intelligence.

In the forensic sample, there were more internalizing problems in non-sexual offenders than in offenders with sexual delinquency only. Group delinquency as opposed to individual delinquency, use of weapons, and severe delinquent acts were all associated with higher depression scores. Finally, self-esteem was lower in juvenile offenders with severe delinquent acts.

Against our expectations and in contrast to previous findings [2,7,8], juvenile offenders reported less internalizing and externalizing problems than clinical patients and rates were comparable to a community sample. Although lower prevalence rates of mental health problems were found in non-detained juvenile offenders compared to a clinical sample of juveniles, half of them had a psychiatric diagnosis. Without forensic referral and assessment, these juveniles would not have been detected and thus would not have received adequate psychiatric treatment.

Previous studies of predominantly detained juvenile offenders found higher rates of psychopathology. Detained juvenile offenders may differ from non-detained juvenile offenders by the higher severity of the crimes they have committed and the presence of psychosocial risk factors for subsequent reoffending (e.g. delinquent peers, lack of parental control). Studies based on incarcerated juveniles found that psychopathology was associated with serious and persistent offending as well as with criminal recidivism [35,36]. In addition, the higher frequencies of psychiatric disorders in detained juveniles may be explained by symptom elevation due to the social isolation and the adversities encountered in the prison environment. The lower rate of psychopathology in non-detained juveniles of the present study may have been associated with less severe crimes and less salient psychosocial risk factors of offending. Thus, not only clinical differences but also differences in the jurisdiction might have played a role because these juveniles were not detained for their offenses.

We found that juvenile offenders showed higher rates of depressive symptoms than community controls. These findings are in agreement with previous studies on depression in detained and non-detained juvenile offenders [10,11,14,15]. In addition and as expected, conduct disorders were more frequently diagnosed in juvenile offenders. However, in contrast to expectations based on a forensic sample, we also observed a high rate of juvenile delinquents without conduct disorders in the present sample. Research has shown that some juveniles commit isolated delinquent acts without a pervasive and persistent pattern of conduct disorder as e.g., reflected in the adolescent limited type of delinquency [37]. Thus, the group of juvenile offenders is heterogeneous regarding to mental health problems and criminal behaviour and some of them, in fact, do not have a psychiatric disorder at all.

The discrepancy between the low rate of self-reported externalizing problems and expert-ratings of conduct disorders in adolescent non-detained offenders is noteworthy. The most plausible explanation for this finding may be selective perception of problems in the non-detained offenders. Confronted with their actual judicial problems including legal consequences, these youngsters may be repressing and denying their behavioural problems in the time frame of the past six months as defined by the YSR. Furthermore, the evaluation took place before they had to appear in court, thus potentially leading to a reduced interest in disclosing any existing behavioural and, in particular, externalizing problems. In addition, the higher rate of borderline intelligence in this sample may have contributed to less self-awareness so that the rate of behavioural problems may have been underreported. Similar conclusions may also be drawn from the study by Vreugdenhil et al. [38] in a sample of incarcerated juveniles who also found that the YSR did not adequately screen for psychiatric disorders. In terms of the clinical implications, forensic experts should be aware of a tendency among juvenile offenders to deny mental symptoms in self-reports. The inclusion of additional informants such as teachers and social workers may be crucial for clinical decision-making in forensic settings.

Although non-detained juveniles had lower rates of clinically diagnosed affective disorders than the clinic sample, the self-reported rates of depressive symptoms in the former group was higher. In terms of severity, these feelings of depression may have been sub-threshold symptoms without fulfilling criteria of a clinical diagnosis. Higher depression scores were observed in some offenders, in particular in group delinquency rather than in individual delinquent acts, in the group of young offenders using weapons, and in the group with more severe delinquent acts. Similar relations of depression and the seriousness of delinquency have also been found by Howard [16]. It has been hypothesized that common risk factors may be responsible for this association of depression and delinquency [39,40]. Furthermore, the depressed juveniles might have actively sought exciting, dangerous, daring or illegal activities in an attempt to relieve their feelings of dysphoria and boredom [41]. Taking our findings of lower self-esteem in the group with severe delinquency into account, one may assume that this offender type might have compensated feelings of inadequacy with delinquent activities with other juveniles. Furthermore, features of irritability might also have played a role in these adolescents with both delinquency and depressive symptoms [9,12]. However, causal relations are not yet clear and depression can also result from criminal behaviour or its consequences such as being arrested [9,42,43] or facing court examination and penal consequences. This explanation may be particularly true for the association of depression and the severity of delinquency: with increasing severity of the crime a more severe punishment has to be excepted so that the depressive reaction may also increase. It might also be a bias of the examination situation as described above, where offenders gladly present themselves as depressive before court.

In contrast to the review by Van Wijk et al. [26], the present study found more self-reported internalizing problems in non-sexual offenders than in sexual offenders. However, it should be noted that the difference was relatively small in magnitude reflecting a difference in less than 3 raw score points and that both scores were clearly in the normal range (equating to standardized T-scores of 50 and 53).

The present study confirms and expands previous findings on the psychopathology in juvenile offenders by focusing on a non-detained sample including to some extent also juvenile offenders with less severe offenses. All these juvenile offenders received forensic assessment on judicial order. Confronted with the information that the expert report of the psychiatric assessment would be given to the court and in the expectation of a juridical decision, these juvenile offenders may have denied their mental problems. Hence, the present findings may differ from those obtained in studies of juvenile offenders in other setting providing strict confidentially of the findings. The present findings may be typical for forensic practice in general and may also be important for clinical decision making in forensic expert reports.

Some limitations of the present study have to be noted. The forensic sample was selective by court decision because not all of the court cases were referred for forensic evaluation. Most frequently, cases with severe delinquency or developmental problems or poor living situations were referred to the forensic unit for further examination or treatment. Furthermore, it has to be acknowledged that self-reports rather than structured clinical interviews were used and that clinical diagnoses were based on expert evaluation using all available information but without formal testing of reliability.

However, the present study also has several strengths. The design used the opportunity to compare a forensic sample with a representative community sample as well as with a clinical sample. The findings support the view that only a subgroup of juvenile delinquents suffer from mental problems and disorders which is in contrast to studies based on incarcerated juveniles with higher rates of psychopathology. Clinicians should be aware of a potential denial of mental symptoms in self-reports of juvenile offenders. The clinical implications for this population include the need of accurate clinical assessment so that the non-detained juvenile offender may also become more aware of his behavioural problems associated with his delinquent acts. Juvenile offenders may benefit from offence-oriented therapy even if psychiatric treatment is not indicated [44].

## Competing interests

None of the authors has any competing financial interests relating to the content of this article.

## Authors’ contributions

DI and HCS designed the present study. HCS was the principal investigator and CWM the research associate of the original Zurich Epidemiological Study of Child and Adolescent Psychopathology (ZESCAP). CB was responsible for the design of the forensic psychiatric documentation system including the offense data system. DI and MA performed the statistical analyses. DI drafted the manuscript and MA, CB, and HCS made substantial contributions to the final manuscript. All authors read and approved the final manuscript.
